# First identification of *Molluscum contagiosum* poxvirus from human in Senegal

**DOI:** 10.4102/jphia.v17i1.1586

**Published:** 2026-05-08

**Authors:** Fatou K. Top, Landry G. Boussiengui, Ndèye C. Sall, Martin Faye

**Affiliations:** 1 Department of Virology, Institut Pasteur de Dakar, Dakar, Senegal

**Keywords:** *Molluscum contagiosum* virus, Senegal, human infection, mpox surveillance, metagenomic sequencing

## Abstract

Herein, we report on the first identification of a human case of *Molluscum contagiosum* virus (MOCV) in Senegal. In 2024, a male child living in Diamniadio, Dakar region, with no history of travel, tested positive for MOCV. The aetiology was identified using metagenomic sequencing in the framework of the ongoing preparedness activities for the 2024 mpox public health emergency of international concern (PHEIC). Given the overlapping clinical features of MOCV infection and mpox, further research on MOCV is warranted in the West African region, particularly in the current context of high mpox circulation.

*Molluscum contagiosum* virus (MOCV) is a double-stranded deoxyribonucleic acid (DNA) poxvirus^1^ that causes a common skin condition that predominantly affects children between 2 years old and 5 years old; however, the infection was also reported in sexually active adolescents and adults with normal immune systems and in immunocompromised individuals of all ages.^2^ *Molluscum contagiosum* virus presents a large genetic diversity with four distinct subtypes (MOCV1–MOCV4), which differ in their genomic composition, geographic distribution and host associations. Among these, MOCV1 is the most prevalent globally, whereas MOCV2 has been increasingly detected in adults, immunocompromised individuals and sexually transmitted infections.^3^ *Molluscum contagiosum* virus infection typically follows a benign and self-limiting clinical course. After an incubation period of 2 weeks to 7 weeks, patients develop small, dome-shaped, umbilicated papules that are usually painless and may appear singly or in clusters on the face, trunk and extremities. Lesions often persist for 6 months to 12 months but may occasionally last up to 4 years, particularly in immunocompromised individuals. Secondary bacterial infection or inflammation may occur when lesions rupture or are scratched. In most cases, spontaneous resolution occurs without scarring as host immunity develops, although treatment may be indicated for cosmetic, discomfort or transmission concerns.^1,2^ Although MOCV has not triggered large outbreaks, it represents a common cause of infectious disease worldwide and has been regularly isolated from humans since the eradication of smallpox.^4,5^

On 21 August 2024, a 5-year-old male living in Diamniadio district, Dakar region, presented to Popenguine healthcare centre with fever, pain and vesicular mucocutaneous rash on the neck. The patient had no history of travel or contact with a known case with pox-like syndrome, and no other systemic symptoms were reported. Oral consent was obtained from the patient’s guardian. Blood and lesion swab samples were collected on the same day and sent to Institut Pasteur de Dakar (IPD) for diagnosis of mpox. The sample tested negative for mpox by quantitative polymerase chain reaction (qPCR) using a multiplex differential assay including mpox, measles, rubella and herpesviruses. The patient was treated with cantharidin, salicylic acid and tretinoin and was discharged on 22 August 2024. The sample was later subjected to a shotgun metagenomic sequencing method, enabling a comprehensive and quantitative assessment of the aetiologies present in a sample.

Interestingly, de novo assembly of generated reads showed a MOCV genome with 80% of coverage (GenBank accession number, MN931752.1). Phylogenetic analysis showed that the newly characterised sequence belonged to subtype 2 MOCV and was closely related to isolates from *Slovenia* and the *United States* between 2017 and 2023 (93%) (Figure 1).

**FIGURE 1:**
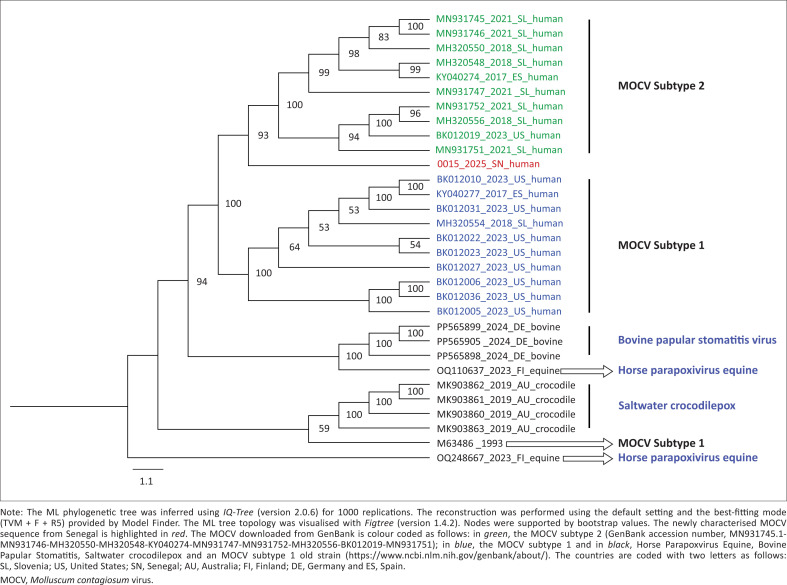
Maximum likelihood phylogenetic tree of the newly characterised *Molluscum contagiosum* virus from Senegal in 2024.

Clinically, there is a challenge in differentiating mpox from infection caused by other poxviruses, such as MOCV, particularly when lesions are multiple or atypical. Our data exhibit the crucial need for differential diagnosis for skin infections during the current mpox outbreak. Although diagnostic resources are limited in many countries in Africa, investment in healthcare and laboratory infrastructures and resources could be prioritised by stakeholders. Continuous clinician training on the potential co-circulation of other skin infections during the current mpox PHEIC and integration of metagenomic sequencing as a second-line testing method in screening of mpox-suspected cases are essential for accurate identification of causative aetiologies. In addition, there is a need for development of specific and affordable assays for the detection of MOCV and promoting more studies integrating genomics and focusing on the assessment of prevalence and dynamics of MOCV in West Africa, especially the subtype MOCV2.

Our data are noteworthy by providing new insights into the epidemiology of poxviruses in West Africa where knowledge on poxviruses is limited. In addition, the newly characterised MOCV2 sequence could be useful in the development of specific diagnostics and therapeutics and in future longitudinal surveillance and experimental studies focusing on understanding MOCV2 molecular epidemiology, pathogenesis and possible transmission dynamics.
